# The prevalence and ossification pattern of the biphalangeal and triphalangeal lateral toes

**DOI:** 10.1007/s00276-018-2027-z

**Published:** 2018-04-17

**Authors:** Marcin Ceynowa, Marek Rocławski, Rafał Pankowski, Tomasz Mazurek

**Affiliations:** Departament of Orthopedic Surgery, Medical University of Gdańsk, ul. Nowe Ogrody 1-6, 80-803 Gdańsk, Poland

**Keywords:** Toe, Foot, Biphalangeal, Triphalangeal, Phalanx

## Abstract

**Purpose:**

Biphalangealism of the toes is an exclusively human phenomenon. The aim of this study was to evaluate the development of the lateral toes in childhood by following the ossification pattern of the phalanges.

**Methods:**

Foot radiographs of 913 adults have been evaluated for biphalangealism of 3rd to 5th toe. The pediatric group, aged 6–15 years of age, was assessed for the number of ossification centers in the foot.

**Results:**

In adults, the mean prevalence of biphalangealism in the 5th toe was 41.39%, in the 4th toe was 2.15%, and in the 3rd toe was 0.48%. In children, 45% feet had four ossification centers in the 5th toe. The epiphysis center of the middle and distal phalanx was missing. In the 4th toe, four centers were present in of 2.47% of cases. Those values are similar to the prevalence of the biphalangeal toes in adult population. The remaining toes had 5 or 6 ossification centers. In the 5-center toe, the epiphysis of the middle phalanx was missing.

**Conclusion:**

A missing distal phalanx epiphyseal ossification center is considered indicative of a biphalangeal toe, and the toes with 5 or 6 ossification centers are indicative of triphalangeal toes. The reason for such evolution of the lateral toes is still debated, but the differences in anatomy most likely have no impact on foot function.

## Introduction

A classical anatomical view of a human foot is a biphalangeal hallux and triphalangeal lesser toes, being a mirror of the human hand. The biphalangeal 5th has been evaluated in several studies and it has been shown that it is common variant [1–4]. However, this information is not widespread among clinicians and can lead to a misdiagnosis, when a fracture of a distal phalanx can be mistaken for a normal triphalangeal 5th toe [5].

Biphalangealism of the toes is an exclusively human phenomenon. The evolution of biphalangeal lateral toes is considered an adaptation to bipedalism, since human is the only bipedal mammal [3]. There even have been attempts to prove that the prevalence of the biphalangeal 5th toe has increased over the last century [4, 6].

The functional relevance of biphalangealism vs triphalangealism has not been proven yet. The aim of this study was to evaluate the development of the lateral toes in childhood by following the ossification pattern of the phalanges.

## Materials and methods

The local hospital radiological database was searched for foot radiographs, performed between January 2016 and August 2017. Included were radiographs from the orthopedic division of the Emergency Department and Department of Orthopedic Surgery. The radiographs were performed for evaluation of trauma or common forefoot pathologies, such as hallux valgus or mallet toes. Exclusion criteria were: fractures and other traumatic pathologies of the forefoot, congenital and developmental foot deformities, advanced osteoarthritis, rheumatoid arthritis, and poor quality of the radiographs.

**Fig. 1:**
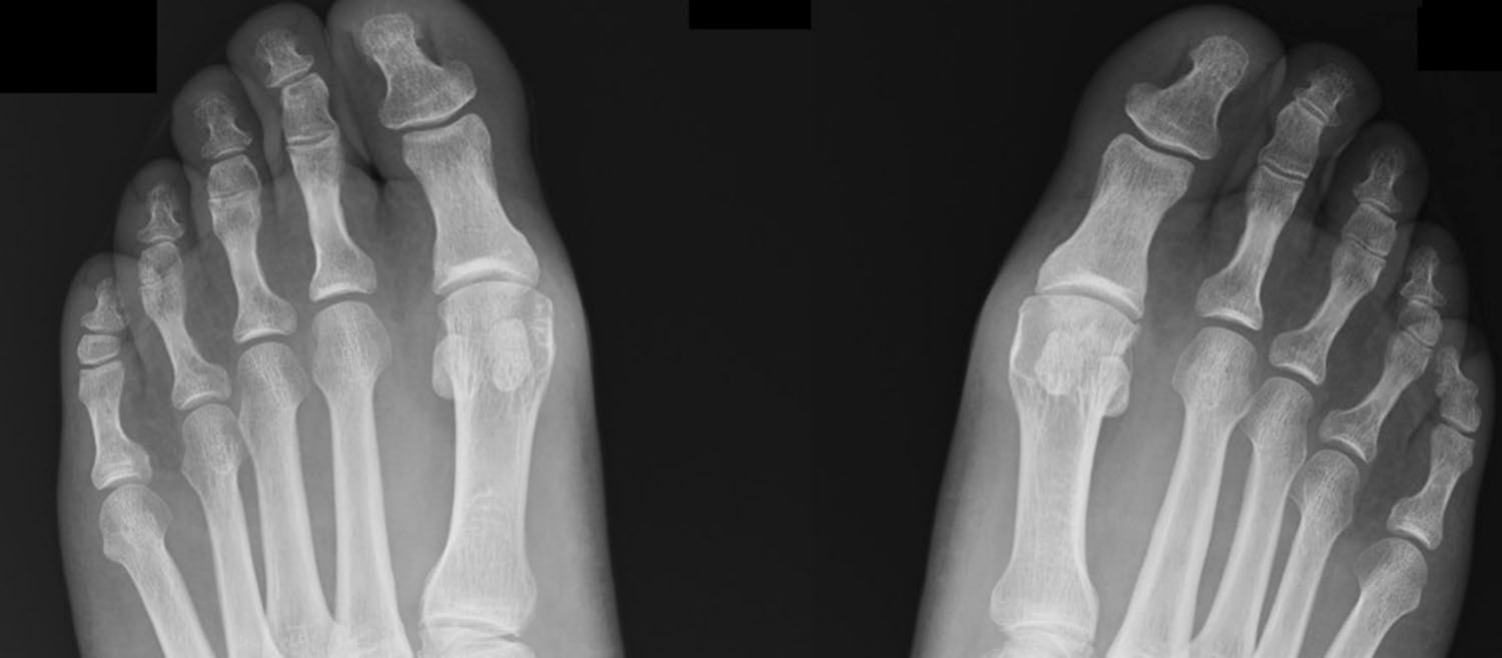
Bilateral dorso-planar foot radiograph of an adult shows a triphalangeal left 5th toe, and a biphalangeal right 5th toe

The adult group included 913 subjects, aged 17–68. Mean age was 41.1 years (SD = 18.3). There were 519 females and 394 males. Bilateral radiographs were available in 63 cases (male/female ratio: 16/47), unilateral in 850 (male/female ratio: 376/474). In the unilateral radiographs, 390 were of the left foot (male/female ratio: 170/220), and 460 of the right foot (male/female ratio: 206/254). All radiographs in this group showed full fusion of the growth plates of the lesser toes. The radiographs were assessed for the number of the phalanges of the lesser toes (Fig. 1).

The pediatric group consisted of 231 children aged 1–14. Pediatric radiographs were divided into groups. In Group I, children were aged 6–14 with closed epiphyseal plates and complete fusion of the ossification centers of the 5th toe. In Group II, children were aged 6–14, with open epiphyseal plates of the 5th toe. In Group III, children were aged 1–5.

Since the main interest of this study lays in the development of the biphalangeal vs triphalangeal 5th toe, fusion was considered complete when middle and distal phalanx of the 5th toe was completely fused. The proximal phalanx is the last to completely fuse, but it is invariably present in all feet, and can be therefore disregarded because it does not influence the variability of the phalanx number [1].

**Fig. 2:**
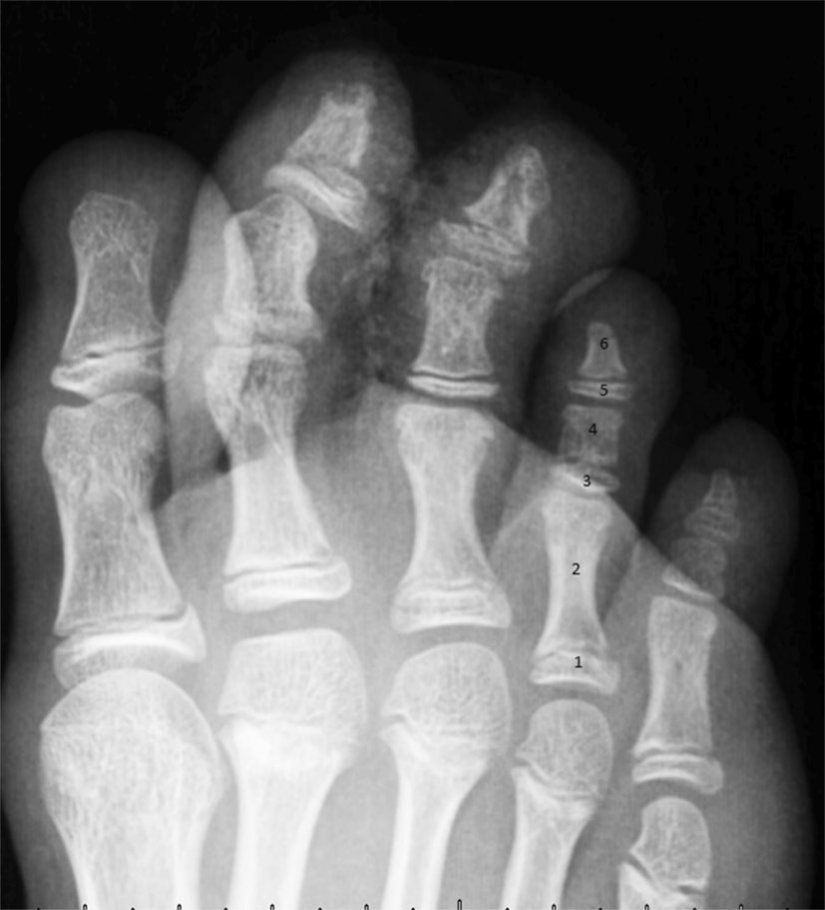
Foot radiograph of a 9-year-old female shows a 5-center 5th toe, and 6-center 2nd–4th toes. The distal phalanx of the 2nd toe is fractured. 1—Proximal phalanx epiphysis; 2—proximal phalanx diaphysis; 3—middle phalanx epiphysis; 4—middle phalanx diaphysis; 5—distal phalanx epiphysis; 6—distal phalanx tuberosity

All children with open plates had the number of ossification centers in 3rd, 4th and 5th toes evaluated. A triphalangeal toe can have a maximum of six ossification centers. In such a case, each phalanx is composed of a proximal epiphysis and a shaft ossification center. The ossification centers were numbered and counted for toes 3–5 (Fig. 2). Children with closed growth plates (Group I) were assessed similar to the adult group.

## Results

### Adult radiographs

Overall, in 976 radiographs (male/female ratio: 411/565), there were 404 biphalangeal 5th toes (male/female ratio: 157/247). The populational ratio is 41.39%. In males it occurs in 38.86%, and in females in 43.71%. Those differences in frequencies were statistically insignificant (*p* = 0.09).

**Fig. 3:**
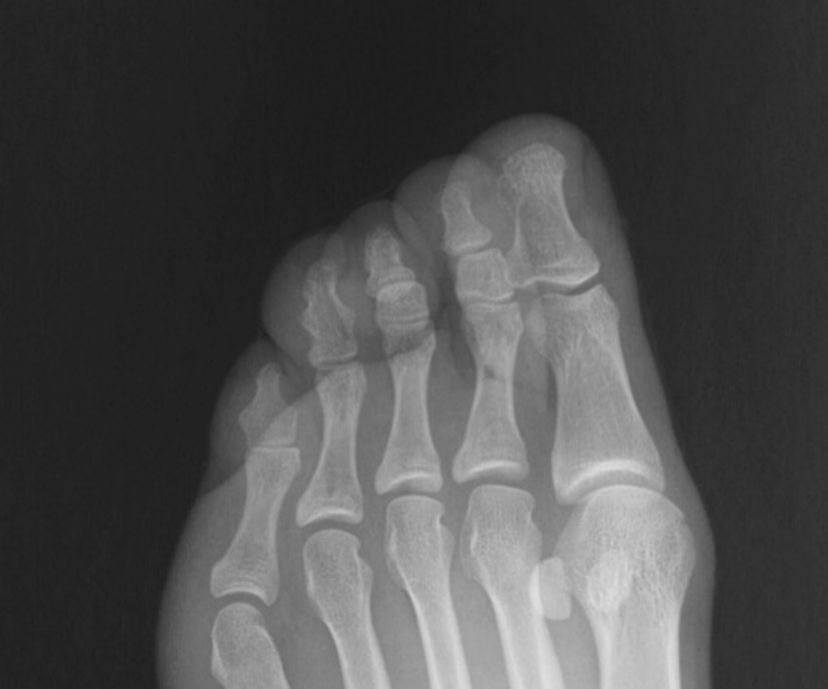
Foot radiograph of an adult shows biphalangeal 4th and 5th toes

In the group with bilateral radiographs (*n* = 63), 20 subjects had bilateral biphalangeal 5th toe (15 females, 5 males). Six subjects had unilateral biphalangeal 5th toe, as shown in Fig. 3, where four biphalangeal were right (one male and three female), and two biphalangeal were left (one male and one female). The remaining 37 had bilateral triphalangeal 5th toe.

The differences of the left/right foot in occurrence of the biphalangeal 5th toe are statistically insignificant (*p* = 0.78).

**Fig. 4:**
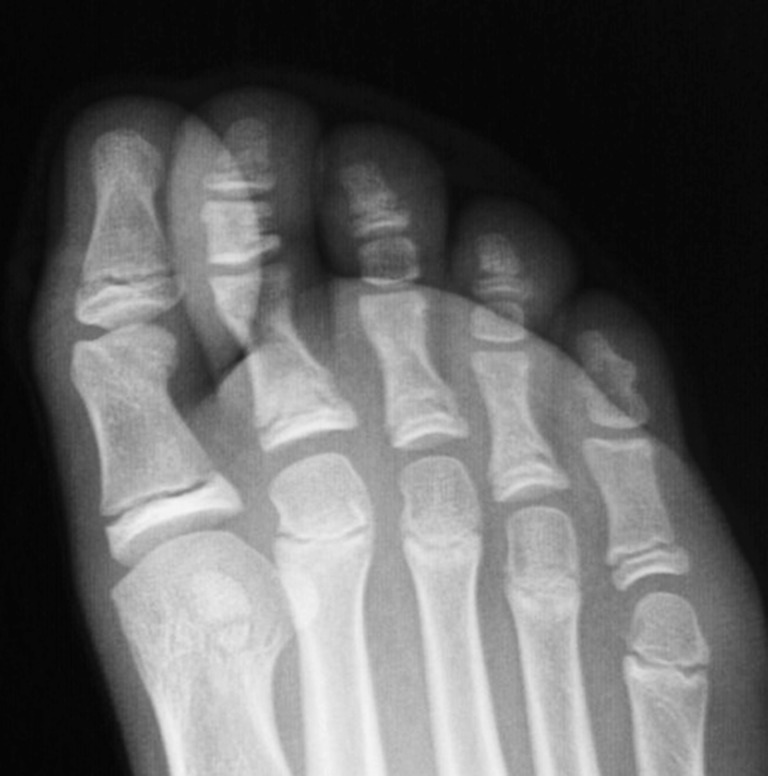
Foot radiograph of a 12-year-old female with complete fusion of the distal phalanx of a biphalangeal 5th toe, and incomplete fusion of the 1st–4th toe

The biphalangeal 4th toe occurs in 21 radiographs (male/female ratio: 5/16), as shown in Fig. 4. The populational ratio is 2.15%. In males it occurs in 1.21%, and in females in 2.83%. Those differences were statistically insignificant (*p* = 0.11).

The biphalangeal 3rd toe occurred in two females (0.48%), and 2nd biphalangeal toe in one female (0.24%).

One female had all her lesser toes biphalangeal (2nd–5th), and one had 3rd, 4th and 5th toes biphalangeal, with the 2nd toe triphalangeal. Both feet were left. All other patients had triphalangeal 2nd and 3rd toes.

The differences in frequencies were compared using Fisher’s exact test. Significance level was 0.05.

### Pediatric radiographs

The radiographs were evaluated for skeletal maturity. The percentage of children with open growth plates in given age is shown in Table 1. All children aged eight and less had open growth plates.

**Table 1:** The percentage of children with open growth plates in given age

Age	Female	Male	Open growth plate—female	Open growth plate—male	Open growth plates total
14 (*n* = 27)	11	16	0 (0%)	6 (37.5%)	6 (22.2%)
13 (*n* = 22)	10	12	1 (10%)	7 (58.3%)	8 (36.4%)
12 (*n* = 33)	19	14	2 (10.5%)	10 (71.4%)	12 (36.4%)
11 (*n* = 34)	16	18	8 (50%)	15 (83.3%)	23 (67.6%)
10 (*n* = 27)	15	12	11 (73.3%)	12 (100%)	23 (85.2%)
9 (*n* = 28)	19	9	16 (84.2%)	9 (100%)	25 (89.3%)
Total (*n* = 171)	90	81	38 (42.2%)	59 (72.8%)	97 (56.7%)

Pediatric radiographs were divided into groups according to fusion of growth plates, as mentioned above. It was observed that some children with fused ossification centers of the 5th toe may have open growth plates in other toes. In group I, eight (10.81%) children in this group had open growth plates of the 4th toe, and of these all had also open growth plates of the 3rd toe. Additional ten children had open growth plates of the 3rd toe, having 4th and 5th toes fused. Totally, 18 children (24.32%) had open growth plates in the 3rd toe. When the 5th toe was fused, the 4th and 3rd growth plates were narrow and it was evident which of the radiological shadows were joints and which were growth plates, as shown in Fig. 4.

In Group II, however, three children had an inverted sequence, with 3rd and 4th toes completely fused, and 5th with open growth plates. All these children were male, and had four ossification centers in the 5th toe, and triphalangeal 4th and 3rd toes.

In this study, fusion of the ossification centers in the lateral toes takes place the period between 9 and 15 years of age.

In Group I (closed growth plates), 26 (male/female ratio: 4/22) out of 74 (male/female ratio: 24/50) subjects had biphalangeal 5th digit, what gives a ratio of 35.13%, similar to the adult ratio. Statistical differences between adults and Group I were insignificant (*p* = 0.32).

In Group II (open growth plates), 57 (male/female ratio: 39/18) out of 125 (male/female ratio: 75/50) subjects had only four ossification centers in the 5th toe, what gives a ratio of 45%, also similar and insignificantly different from both the adult group (*p* = 0.38) and pediatric group with fused ossification centers (*p* = 0.18). The remaining children in this group had five ossification centers in the 5th toe.

In this group, 4 (M/F: 2/2) children of 122 with unfused 4th toe had only four ossification centers in this toe, what gives a ratio of 2.47%, similar to the adult ratio for biphalangeal 4th toe. From the remaining 118 children with unfused 4th and 5th toes, 95 (80.5%) had five ossification centers and 23 (19.5%) had six centers in the 4th toe, what probably corresponds with different ossification patterns of a triphalangeal 4th toe.

In the 3rd toe, all children in Group II had either five (72 children, 59.01%) or six ossification centers. All children with six ossification centers in the 4th toe had also six ossification centers in the 3rd toe, but some children with six centers in the 3rd toe had five centers in the 4th.

**Fig. 5:**
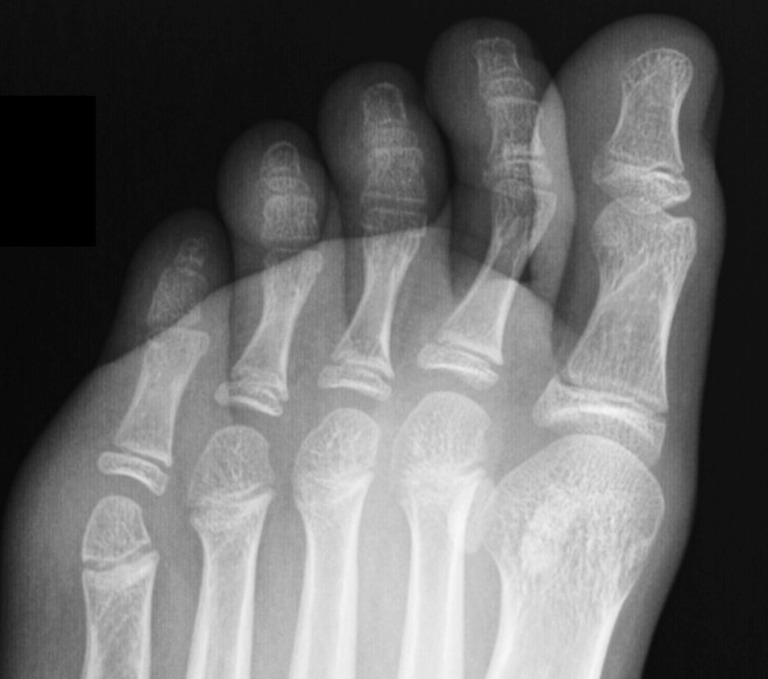
Foot radiographs of a 11-year-old female shows a 4-center 5th toe, before fusion into biphalangeal 5th toe

Most children with four centers in the 5th toe had five centers in the 4th; however, six (4.8%) children had six centers in both other toes (Fig. 5). All children with four centers in the 4th toe had four centers in the 5th toe. Moreover, three of them had five centers in the 3rd toe, but one had six centers in the 3rd toe. The ossification pattern in this group was invariably as shown in Table 2.

**Table 2:** Ossification pattern of the lesser toes

Phalanx	Ossification center name	Center number (Fig. 4)	4-center toe	5-center toe	6-center toe
Proximal phalanx	Proximal epiphysis	1	(+) Present	(+) Present	(+) Present
	Shaft	2	(+) Present	(+) Present	(+) Present
Middle phalanx	Proximal epiphysis	3	(−) Missing	(−) Missing	(+) Present
	Shaft	4	(+) Present	(+) Present	(+) Present
Distal phalanx	Proximal epiphysis	5	(−) Missing	(+) Present	(+) Present
	Tuberosity	6	(+) Present	(+) Present	(+) Present

In Group III, in children aged 1–5, the ossification centers were present, but its number varied (Table 3).

**Table 3:** The number of ossification centers in lateral lesser toes in Group III

Age	5th toe	4th toe	3rd toe
2 (*n* = 9)	3 (*n* = 1)	4 (*n* = 5)	4 (*n* = 3)
	4 (*n* = 6)	5 (*n* = 3)	5 (*n* = 6)
	5 (*n* = 2)	6 (*n* = 1)	
3 (*n* = 10)	4 (*n* = 9)	4 (*n* = 7)	4 (*n* = 2)
	5 (*n* = 1)	5 (*n* = 3)	5 (*n* = 7)
			6 (*n* = 1)
4 (*n* = 8)	4 (*n* = 3)	5 (*n* = 5)	5 (*n* = 5)
	5 (*n* = 5)	6 (*n* = 3)	6 (*n* = 3)
5 (*n* = 5)	4 (*n* = 3)	4 (*n* = 1)	4 (*n* = 1)
	5 (*n* = 2)	5 (*n* = 4)	5 (*n* = 4)
			6 (*n* = 5)
Total (*n* = 32)	3 (*n* = 1)	3 (*n* = 0)	3 (*n* = 0)
	4 (*n* = 21)	4 (*n* = 13)	4 (*n* = 6)
	5 (*n* = 10)	5 (*n* = 15)	5 (*n* = 21)
	6 (*n* = 0)	6 (*n* = 4)	6 (*n* = 5)

In all children in Group II and III, the 4th toe always had a greater or equal number of ossification centers as the 5th toe. The same pattern was observed regarding the 4th and 3rd toe.

## Discussion

The findings of the overall prevalence of biphalangeal 3rd, 4th and 5th toes have been similar to other studies that evaluate Caucasian population [1, 2, 7, 8]. In this study, biphalangeal 5th toe was found in 38.86% in males, and in females in 43.71%. This difference was statistically insignificant, as in most other studies [1, 2, 4]. However, in a study by Gallart et al. [4], the differences between sexes were significant: biphalangeal 5th toe was present in 39.88% in men, and in 47.35% in women.

No significant differences were found between left and right feet, as in other studies [1–4].

In bilateral radiographs, a minority of patients had a biphalangeal 5th toe in one foot, and a triphalangeal in the other foot, which is also confirmed in other studies [8]. In our study, the incidence of asymmetry (6/63, 9.5%) is high when compared to other studies (2.6% in Gallart et al. [4]), but our sample is relatively small and should be considered with caution.

The comparison between adult and pediatric population has been made to determine the developmental ossification pattern of the phalanges. It is reasonable to assume that different number of ossification centers may be responsible for a different final number of phalanges.

The ossification centers appear and fuse in different periods of childhood [1, 9–11], and this process may vary individually quite significantly. Children younger than 6 years in this study, as well as in other, were evaluated as a separate group, since in this age not all ossification centers might have appeared and the results may be therefore biased.

In our study, we evaluated the prevalence of ossification centers in pediatric population, and found that ossification follows a specific pattern. There were three patterns observed, including 4, 5 or 6 ossification centers (Table 2). Other patterns of ossification were not found. Moreover, in older children, it was often obvious which toe will eventually have two or three phalanges, because the shape of the centers before fusion was identical to adult phalanges, apart from a narrow shadow of the growth plate (Figs. 4, 5). The ossification patterns as mentioned above, correspond with other studies [1].

When the findings were compared with the adult population, we found the prevalence of 4-center ossification pattern to be similar to the occurrence of biphalangeal toes. The 5- and 6-center pattern combined is similar to the occurrence of the triphalangeal toe. This is true for both 5th and 4th toes. Also, in older children, where the phalanx shape was clearly recognizable, the number of phalanges always matched the above-mentioned patterns.

We believe that the 4-center toe, which includes the absence of the secondary ossification center of the proximal epiphysis of the distal phalanx, is a radiological marker of the development of a biphalangeal toe (Fig. 5).

The 5- and 6-center toes are indicative for the development of triphalangeal toe (Fig. 2). The ossification pattern is variable only regarding the middle phalanx, which can develop with a single ossification center (primary diaphyseal center) or with two centers, the primary diaphyseal and secondary epiphyseal centers. The occurrence of the epiphyseal center is variable, however, a pattern can be found: it occurs with decreasing frequency from medial to lateral toes, and is never found in the 5th toe. Those findings correspond well with more recent study [1], they differ, however, with classic study by Venning [11].

It must be noted that this conclusion is based on statistical data. It could be proven only by performing foot radiographs throughout childhood every few years, in a large group of subjects, to observe the development of ossification centers until final fusion of the phalanges. Such a study done for sole scientific purposes is impossible to perform without compromising ethical standards. Radiographs of normal feet in children are performed in practice only after a traumatic event, and therefore such a series is extremely difficult to find. Foot radiographs are performed on a regular basis to guide treatment in cases of foot deformity, what is an exclusion criterion for such a study.

A similar pattern of laterally decreasing number of ossification centers is found in children younger than five (Table 3), but as mentioned before, not all secondary ossification centers may yet be visible on radiographs and their final number should not be considered until later in life.

However, studies that evaluate the development of the phalanges in neonates evaluate the presence of ossification centers.

It is well known that radiographic image of a bone in childhood differs greatly from the actual shape of the bone. The “bone” is composed mainly of cartilage, which turns into ossified bone in a precise sequence of ossification of primary and secondary ossification centers, which are separated by growth cartilage [9]. The development of two or three joints in the toes is determined genetically [4]. The joints are formed intrauterine, and the once formed joints cannot be fused because of failure of an ossification center to appear, or its development cannot be halted by shoe wearing, as suggested by some [12]. On the other hand, however, possibly the number of ossification centers may be influenced by the presence or absence of a joint.

The fusion of the growth plates usually follow a pattern: the lateral toes are the first to fuse. The more medial the toe is, the later the fusion occurs. However, in the current study, a few cases of a medial to lateral sequence of fusion were found. The lateral to medial pattern may be a result of the smaller size of the phalanges of the lateral toes and the tendency for a smaller number of growth plates. Therefore, when the bone matures at a constant rate in the growth plate, the bone requires less time to reach its final size [9, 10].

Since 5th toe biphalangealism is an exclusively human phenomenon, it is postulated that it is an adaptation to bipedalism [3]. The evolutionary reduction of the number of phalanges has been difficult to prove. Studies that compare past populations modern ones show conflicting results: a study between populations [6] shows increased prevalence of biphalangealism in younger population, but another study [4] found an increase in biphalangealism of the 4th toe in modern population as compared to previous studies from 1905, but a decrease of its prevalence in the 5th toe.

It should be noted, however, the above mentioned studies span between only 50 and 150 years. Significant adaptive changes in the skeleton take millions of years rather than hundreds [13]. One should take into consideration that if the number of lesser toe phalanges offered significant advantages or disadvantages in walking or running, one of those variants would have been virtually eliminated when early humankind was walked barefooted, and not in the age of shoes, cars and public transport.

The actual advantages or disadvantages of biphalangeal over triphalangeal toes have not been yet proved. Some light might be shed on the issue through studies that evaluate the correlation of biphalangealism vs triphalangealism and forefoot pathology. Gallart showed that a triphalangeal 5th toe is associated with higher prevalence of forefoot pathology, which would support the thesis that biphalangeal individuals are better adapted to bipedalism [8]. However, others found an increased prevalence of forefoot pathology increased in feet with biphalangeal 5th toes [7, 14].

The reason for such evolution of the lateral toes is still debated. One of the reasons may be that the importance of the toes seems to decrease from medial to lateral. While the hallux is crucial for correct walking pattern, and its strength and mobility is of utmost importance, the loss of joint mobility, arthritic changes or even amputation of the lesser toes do not pose such a significant disability for the patient [15].

The importance of individual toes in standing and walking decreases from medial to lateral. The 5th toe is not in any contact with the ground in about a 1/3 of people in standing, and during walking, it has the shortest contact time with the ground, as compared with other toes. About 60% of peak contact force of the toes with the ground during walking is transferred through the great toe, and only 5–6% through the 5th toe, with the rest gradually divided between the remaining toes [15]. This fact shows that the more lateral the toe is, the lesser its importance in bipedal walking. The differences in anatomy of the 5th and perhaps 4th toe, even as substantial as loss of one joint, most likely have no impact on foot function. Therefore, the lateral lesser toes are subjected to smaller evolutionary pressure, hence its increased anatomical variability in humans.

To sum up, the prevalence of ossification centers in the lateral lesser toes is variable, but follows a pattern. The number of centers in the more lateral toe may be the same or less than the adjacent medial toe. In a toe, at least four ossification centers appear, they are invariably: primary ossification centers of the proximal and middle phalangeal diaphysis and distal phalangeal tuberosity, as well as the proximal secondary phalanx ossification center (epiphyseal). The middle phalanx secondary ossification center (epiphyseal) is always missing in the 5th toe, and occurs with variable prevalence in the remaining lesser toes. The presence or absence of the middle phalanx epiphysis seems not to be indicative of the final number of the phalanges. The presence of the distal phalanx base ossification center (epiphysis) in children corresponds well with the prevalence of biphalangealism of the corresponding toe in adults. Therefore, it should be considered indicative of the final number of phalanges in adulthood.
